# How Is CYP17A1 Activity Altered in Autism? A Pilot Study to Identify Potential Pharmacological Targets

**DOI:** 10.3390/life12060867

**Published:** 2022-06-10

**Authors:** Benedikt Andreas Gasser, Johann Kurz, Bernhard Dick, Markus Georg Mohaupt

**Affiliations:** 1 Departement für Bewegung und Sport, Universität Basel, 4001 Basel, Switzerland; 2 Intersci Research Association, Karl Morre Gasse 10, 8430 Leibnitz, Austria; john@a1.net; 3 Department of Nephrology and Hypertension, University of Bern, 3012 Berne, Switzerland; bernhard.dick@gmx.ch; 4 Teaching Hospital Internal Medicine, Lindenhofgruppe, 3006 Berne, Switzerland; markus.mohaupt@lindenhofgruppe.ch

**Keywords:** DHEA, androsterone, etiocholanolone, tetrahydrocorticosterone

## Abstract

**Background:** Increasing evidence exists that higher levels of androgens can be found in individuals with autism. Evidence yields to a susceptible role of Cytochrome P450 17A1 (CYP17A1) with its catalyzation of the two distinct types of substrate oxidation by a hydroxylase activity (17-alpha hydroxylase) and C17/20 lyase activity. However, to what extent steps are altered in affected children with autism versus healthy controls remains to be elucidated. **Methods:** Urine samples from 48 boys with autism (BMI 19.1 ± 0.6 kg/m^2^, age 14.2 ± 0.5 years) and a matched cohort of 48 healthy boys (BMI 18.6 ± 0.3 kg/m^2^, 14.3 ± 0.5 years) as well as 16 girls with autism (BMI 17.5 ± 0.7 kg/m^2^, age 13.8 ± 1.0 years) and a matched cohort of 16 healthy girls (BMI 17.2 ± 0.8 kg/m^2^, age 13.2 ± 0.8 years) were analyzed for steroid hormone metabolites by gas chromatography-mass spectrometry. **Results:** The activity of 17-alpha Hydroxylase increased by almost 50%, whereas activity of 17/20 Lyase activity increased by around 150% in affected children with autism. Furthermore, the concentration of Cortisol was higher as compared to the average increase of the three metabolites TH-Corticosterone, 5α-TH-Corticosterone and TH-11β-DH-Corticosterone, indicating, in addition, a stimulation by the CRH-ACTH system despite a higher enzymatic activity. **Discussion:** As it was shown that oxidative stress increases the 17/20-lyase activity via p38α, a link between higher steroid hormone levels and oxidative stress can be established. However, as glucocorticoid as well as androgen metabolites showed higher values in subjects affected with autism as compared to healthy controls, the data indicate, despite higher CYP17A1 activity, the presence of increased substrate availability in line with the Cholesterol theory of autism.

## 1. Introduction

**Figure 1:**
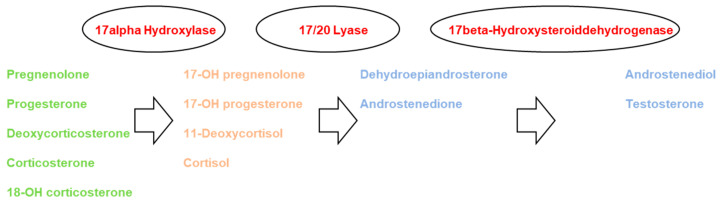
Steroid hormone synthesis pathway with the two CYP17A1-dependent steps of 17alpha-Hydroxylase and 17/20 Lyase activity [4,14,28]. Green (mineralocorticoids), orange (glucocorticoids), blue (androgens).

Already, in one of the first description of children with autism from Hans Asperger in 1944, altered steroid hormones were implied [1]. Hans Asperger showed four cases of children with autism, whereby in one child, definitely dysregulated steroid hormones were present, implied by the clinical description [2]. In consequence, lots of efforts were performed to elucidate the endocrine phenotype of this disorder. Special attention was first paid to the HPAG-axis with a special interest in the CRH-ACTH system (reviewed by Taylor and Corbett [3]). Theories such as extreme male theory [4] or the Cholesterol theory of autism [5] were directly aiming at steroid hormones. Further supporting relevance can be derived that altered sex hormones are in line with the around four times higher prevalence of autism in boys as compared to girls [4,5]. A parallel with the increasing evidence of altered steroid hormones genetic studies were performed, indicating, for example, an association with fragile X-Syndrome in patients with autism in line with altered androgens [6]. Furthermore, a direct association was performed between autism and RORA as a novel candidate gene in autism, directly implying a role of Cytochrome P450 17A1 (CYP17A1; also P450c17 and P450sccII) [7,8,9,10,11]. Besides the mentioned lines of evidence, dysregulated steroid hormones were descriptively measured several times in boys as well as girls with autism [12,13]. Measurements from urine [12,13], plasma [14] or amniotic fluid [4] were performed and alterations were detected in pre-pubertal, as well as post-pubertal boys and girls. A recent systematic review and metanalysis from a respectable number of 321 boys and 64 girls with autism indicated that 17/20 Lyase activity is altered in boys as well as girls, implying a relevant role of CYP17A1 [15]. CYP17A1 is a critically important enzyme in humans that catalyzes the formation of all endogenous androgens via two steps: the 17alpha Hydroxylase step and the 17/20 Lyase step [9] (Figure 1). Through its hydroxylase activity, it catalyzes the 17alpha-hydroxylation of pregnenolone to 17alpha-OH pregnenolone [9]. Subsequently, through its C17/20lyase activity, it can further convert 17α-OH pregnenolone to the androgen dehydroepiandrosterone (DHEA), which is a precursor to androstenedione, testosterone and dihydrotestosterone [9,16] (Figure 1). To date, more than 100 mutations in the CYP17A1 gene have been described and the majority are associated with a classic phenotype of combined 17alpha-hydroxylase and 17/20-lyase deficiency [17,18,19,20,21,22]. Interestingly, the reduction of 17alpha-hydroxylase activity and/or 17/20 Lyase activity seems to be a mirror image of the alterations of the steroid hormones detected in affected subjects with autism [4,12,13,14,15,23,24]. One key question is whether CYP17A1 stops after 17α-hydroxylation or proceeds to 17/20 lyase activity, which is largely dependent on three post-translational factors [9,16]. First, 17/20 lyase activity is especially sensitive to the molar abundance of the electron-transfer protein P450 oxidoreductase (POR) [9,16]. Second, cytochrome b5 strongly promotes 17/20 lyase activity, principally by acting as an allosteric factor promoting the interaction of CYP17A1 with POR [9,16,25,26]. Third, the serine/threonine phosphorylation of CYP17A1 itself promotes 17/20 lyase activity, again apparently by promoting the interaction of CYP17A1 with POR [9,16]. The principal kinase that phosphorylates CYP17A1 to confer 17/20 lyase activity appears to be p38α (MAPK14), which increases the maximum velocity of the 17/20 lyase reaction, while having no detectable effect on the 17alpha-hydroxylase reaction [9,16].

To point out, based on different lines of evidence, a role of CYP17A1 seems crucial in autism, however, the direct description of causes and mechanisms is still missed. In this work, we try to put the focus on CYP17A1 activity and try to elucidate the roles of the two enzymes 17-alpha Hydroxylase and 17/20 Hydroxylase by comparing measurements of the relevant steroid hormones indicating potential differences in the activity of CYP17A1 in affected children with autism versus healthy controls. As this is a hypothesis with potential for falsification, it shall be stated that CYP17A1 activity is not altered in children with autism versus healthy controls [27].

## 2. Material & Methods

### 2.1. Participants

Forty-eight boys with autism (BMI 19.1 ± 0.6 kg/m^2^, age 14.2 ± 0.5 years) and a cohort of forty-eight individually-pairwise healthy boys were matched by BMI and age (BMI 18.6 ± 0.3 kg/m^2^, 14.3 ± 0.5 years), as well as 16 girls with autism (BMI 17.5 ± 0.7 kg/m^2^, age 13.8 ± 1.0 years) and a cohort of 16 individually-pairwise healthy girls matched by BMI and age (BMI 17.2 ± 0.8 kg/m^2^, age 13.2 ± 0.8 years). Individuals were included after they had given written informed consent which was also signed by their legal guardians. In the control group, autism was excluded by the Marburg questionnaire for Asperger syndrome (MBAS), as completed by the caregivers. The study was approved by the governmental ethics board of Graz, Austria (Approval Number FA8B-50.2), and registered at ClinicalTrials.gov ID NCT01197131.

### 2.2. Study Procedure

Children with autism and healthy control girls were recruited from the area of Leipzig (Austria). Enrolment took place from mid-2009 to mid-2012. All participants were Caucasians. Participants were excluded if they had a history of liver diseases, renal or endocrine disorders, a current infection or fever. Intellectual disability or behavioral disorders were exclusion criteria only for the control group, but were allowed as comorbid conditions in the group of children with autism, whereby one girl had to be categorized as intellectually disabled. The diagnosis was given in the first years of the children’s lives according to the diagnostic criteria of the DSM-IV (for details, see Supplementary Table S1) and was cross-checked by experienced clinicians (i.e., medical doctors and/or psychologists) during enrolment of the study (for details of diagnosis, see Supplementary Table S1). All procedures performed in the studies involving human participants were in accordance with the ethical standards of the national research committee and with the 1964 Helsinki Declaration and its later amendments. The study was approved by the governmental ethics board of Graz, Austria, and registered at ClinicalTrials.gov. Involvement in the study was voluntary and not compensated.

## 3. Methods

Urine samples were taken between 7 and 9 a.m. in the morning after breakfast. Urine sample preparation comprised pre-extraction, enzymatic hydrolysis, extraction from the hydrolysis mixture, derivatization and gel filtration, as previously described [29,30,31]. The recovery standard was prepared by adding 2.5 µg of medroxyprogesterone to 1.5 mL of urine. The sample was extracted on a Sep-Pak C18 column (Waters Corp., Milford, MA, USA), dried, reconstituted in 0.1 M acetate buffer, pH 4.6, and hydrolyzed with powdered Helix pomatia enzyme (12.5 mg; Sigma Chemical Co., St. Louis, MO, USA) and 12.5 µL of β-glucuronidase/arylsulfatase liquid enzyme (Roche Diagnostics, Rotkreuz, Switzerland). The resulting free steroids were extracted on a Sep-Pak C18 cartridge. A mixture of internal standards (2.5 μg each of 5α-androstane-3α, 17α-diol, stigmasterol and cholesterol butyrate, and 0.15 μg of 3β5β-tetrahydroaldosterone) was added to this extract and the sample derivatized to form the methyloxime–trimethylsilyl ethers. Analyses were performed on a Hewlett-Packard gas chromatograph 6890 (Hewlett Packard, Palo Alto, CA, USA) with a mass selective detector 5973 by selective ion monitoring. One characteristic ion was chosen for each compound measured. The derivatized samples were analyzed during a temperature-programmed run (210–265 °C) over a 35 min period. The calibration standard consisted of a steroid mixture containing known quantities of all steroid metabolites to be measured. Responses and retention times were recorded regularly. In each case the ion-peak was quantified against that of the stigmasterol internal standard. All steroid hormone metabolites measured were corrected for urinary creatinine excretion. Apparent enzyme activities were calculated as ratios of the relevant metabolites measured (Androsterone, Etiocholanolone, DHEA, Androstendione, Testosterone, TH-11β-DH-Corticosterone, TH-Corticosterone, 5α-TH-Corticosterone and Cortisol), as previously described by us and others [29,30,31].

## 4. Statistical Analysis

Descriptive statistics were calculated with mean and SEM for all metabolites. In addition, all metabolites were analyzed concerning the normal distribution with the Jarque–Bera test. A hypothesis of the normal distribution of all of the sets of the measured values could not be rejected, at least on the alpha = 0.10 level, except for DHEA and tetrahydrocorticosterone in girls with autism [32]. Therefore, two-sided paired t-tests with a Bonferroni correction for multiple comparisons were performed for the metabolite classes, except for DHEA and tetrahydrocorticosterone where Wilcoxon Tests were performed due to the missing normal distribution. Data are presented as a mean ± SEM. Analyses were conducted with Graphpad Prism (GraphPad Software, Inc., La Jolla, CA, USA), Microsoft Excel (Microsoft Inc., Redmond, WA, USA) and SPSS (IBM Inc., Armonk, NY, USA).

## 5. Results

**Figure 2:**
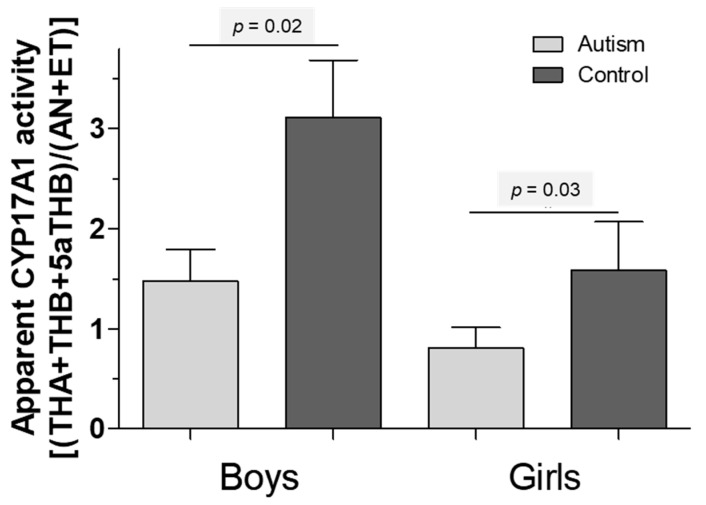
A low ratio of tetrahydro-11β-dehydrocorticosterone + tetrahydrocorticosterone + 5α-tetrahydrocorticosterone to androsterone + etiocholanone ((THA + THB + 5α-THB)/(AN + ET)), suggesting an increased cytochrome P450 17A1 (CYP17A1) activity. (Grey: children with autism, black: healthy children).

Despite thorough age, BMI and gender matching, with no significant differences for age and BMI in the cohort of affected versus healthy children, the urinary secretion of almost all steroid hormone metabolites tended to be altered in children with autism, reaching statistical significance more frequently in boys versus girls (for details, see previous analyses [12,13]). Using the assumption of a general dysregulation of steroid hormones as a starting point to elucidate the role of CYP17A1 activity in depth, Table 1 summarizes the steroid hormones of interest for a sample of 48 boys with autism versus an individually-pairwise matched cohort of healthy boys and, in addition, 21 girls with autism versus an individually-pairwise matched cohort of healthy girls. When focusing on activities of 17-alpha Hydroxylase and 17/20 Lyase, we refer to Figure 2 which tries to capture one aspect of excessive androgen production in autism. It is indicated by a low ratio of tetrahydro-11β-dehydrocorticosterone + tetrahydrocorticosterone + 5α-tetrahydrocorticosterone to the sum of androsterone + etiocholanone ((THA + THB + 5α-THB)/(AN + ET)), suggesting increased cytochrome P450 17A1 (CYP17A1) activity (Figure 2).

**Figure 3:**
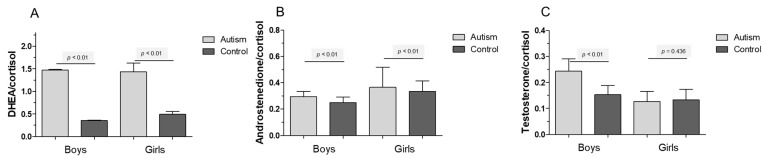
(**A**) Ratios of dehydroepiandrosterone (DHEA) to cortisol in either control (dark grey bars) or children affected by ASD (light grey bars) for (**B**) Androstenedione/Cortisol and (**C**) Testosterone/Cortisol. High ratios in autistic children suggest a preference for DHEA over cortisol. Means ± SEM are given [33].

Figure 3 encompasses the ratios of Cortisol to DHEA (Figure 3a), Cortisol to Androstenedione (Figure 3b) and Cortisol to Testosterone (Figure 3c). DHEA and cortisol characterize the main metabolites following 17α-hydroxylation towards either 11β- or further 20α-hydroxylation. The metabolism in children with autism is clearly shifted towards DHEA (*p* < 0.01). Increased ratios of testosterone to Cortisol can be detected when comparing boys with autism versus healthy controls (*p* < 0.01). Ratios of Androstenediol to Cortisol show about a double value for boys with autism (*p* < 0.01).

**Figure 4:**
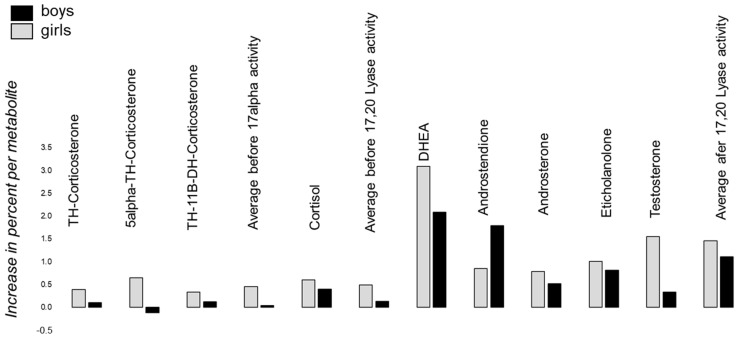
The percent increase or decrease in boys (black) and girls (grey) with autism versus healthy controls per metabolite is shown. The ratios were calculated by dividing the mean concentration of a metabolite of boys and girls with autism, respectively, by the mean concentration of healthy boys and girls, respectively. The increase in Cortisol is somewhat higher than the average before 17-alpha activity, indicating that, in addition, a stimulation of HPAG via CRH-ACTH is present in affected subjects with autism.

Figure 4 tries to capture the percent increase or decrease in boys and girls with autism versus healthy controls per metabolite. The ratios were calculated by dividing the mean concentration of a metabolite of boys and girls, respectively, with autism by the mean concentration of healthy boys and girls, respectively. Despite 5α-TH-Corticosterone in girls, the average metabolite levels are always higher in affected boys and girls with autism, respectively. By deciphering the information in detail, the concentration of Cortisol is higher compared to the average increase of the three metabolites TH-Corticosterone, 5α-TH-Corticosterone and TH-11β-DH-Corticosterone, indicating an increased 17-alpha activity and/or a higher stimulation by the CRH-ACTH system, as Cortisol is directly released by ACTH in the adrenal gland (Figure 4). The increase before was around 50% and almost the same before and after 17-alpha activity (Figure 4). Furthermore, in boys as well as girls, the average of the ratio of metabolites after 17/20 Lyase activity shows an increased activity of around 150%. In consequence, both catalyzing steps imply an increased activity of 17-alpha Hydroxylase and 17,20 Lyase, whereby the activity of 17/20 Lyase is more affected than 17-alpha Hydroxylase.

## 6. Discussion

The aim of this study was to analyze potential alterations of the activity of CYP17A1 in affected boys and girls with autism versus healthy controls. The initially stated hypothesis, that there is no difference in the activity levels of CYP17A1 in children with autism versus healthy controls, seems to be wrong. As previously indicated by us and others, a dysregulation with a general dysregulation not solely of CYP17A1, but in general (not only androgens), is most conspicuous in boys and girls with autism [4,12,13,14]. Here, we focused in detail on the role of 17alpha Hydroxylase and 17/20 Lyase activity. Generally, as cortisol, as well as testosterone, shows an almost double value of steroid hormone concentrations in boys with autism as compared to the healthy cohort, a general pattern of a substantial increase in glucocorticoids and androgens seems to exist, allowing the suggestion that a principal overload of steroid metabolites is present in affected children with autism. This finding is in line with the Cholesterol Theory of autism, as cholesterol is the main precursor of steroid hormones [5] (Figure 1). In addition, it would explain the increased release of ACTH through stimulation on the level of the central nervous system by CRH (reviewed by Taylor and Corbett [3]). ACTH is a strong regulator of VEGF and, in consequence, Angiogenesis, which implies a number of effects on the nerve cell level supplying the paradigm of autism as a pervasive disorder [34,35]. However, we further believe that the disturbed HPAG-axis with an increased substrate availability is neither solely causative, nor the main cause of autism. This is indicated by the fact that the ratio of DHEA to cortisol is displaced towards the DHEA, suggesting a preference of routing precursors towards androgens instead of cortisol. Mechanistically, such conditions have been described as enhancing coenzyme or electron transfer availability via stimulated oxidoreductase activity to excess androgen exposure despite otherwise normal regulatory feedback responses [36]. This is conceivable in conditions of inherited stress susceptibility such as, for example, preterm birth, in induced stress conditions and during developmental periods with an inevitably high stimulatory impact on androgen production [36,37,38,39]. Nevertheless, alterations on the level of 17-alpha Hydroxylase and 17/20 Lyase and, in addition, the CRH-ACTH System are in line with the findings that the average increase of metabolites measured prior to 17-alpha Hydroxylase activity (TH-Corticosterone, 5α-TH-Corticosterone and TH-11β-DH-Corticosterone) is less increased in affected children with autism as compared to the increase of Cortisol, indicating, in addition, stimulation by the CRH-ACTH system (Figure 4). When focusing on the specific cascades altered, it becomes evident that both steps, the 17-alpha Hydroxylase and the 17/20 Lyase step, seem to be altered; however, the second step is more pronounced compared to the first (Figure 4). The activity of 17-alpha Hydroxylase increased by almost 50%, whereas the activity of 17/20 Lyase increased by around 150%. However, when reflecting the above-mentioned points we might forget the multiple phenotypes of autism, which might have an impact on the degree of alteration of CYP17A1 activity. For example, in our samples we had boys and girls with the previous diagnosis of DSM-IV of Kanner Syndrome, Asperger Syndrome and Atypical Autism (Supplementary Table S1). Nowadays it is well known that these diagnoses are not used anymore for different reasons, and it is simply spoken about autism spectrum disorders. In consequence, different degrees of social and communicative impairment are subsumed under one diagnosis. Thereby, the degree of social and communicative impairment might be correlated with the alterations of CYP17A1 activity. This aspect is not captured by our study design limiting the findings in consequence. Maybe in further studies it might be interesting to correlate the severity of the autism with CYP17A1 activity and to develop a cut-off of alteration, as this would allow one to implement a rational pharmacotherapy.

When searching for potential treatment options in autism, pharmaceuticals directly addressing CYP17A1 activity—especially 17/20 Lyase activity—should be considered as therapeutical targets. These pharmaceuticals, for example, abiraterone, are mainly known for the treatment of prostate cancer to reduce androgen concentrations [40,41,42]. Furthermore, Metformin might be an opportunity which is used for the metabolic consequences seen in polycystic ovary syndrome for its androgen-lowering and insulin-sensitizing properties [36]. Interestingly, the approach to use insulin-sensitizing drugs for affected subjects with autism is not new [43] and it was shown in a mouse model of autism that Metformin showed positive effects on social behavior in C57/BL6 mice [44]. Furthermore, Metformin was already used in the autistic context to lower weight gain due to antipsychotic therapy [45]. Yet, as well as for abiraterone the mechanism of action of Metformin remains unclear [46]. Two potential targets for metformin regulating steroid and glucose metabolism are AMP-activated protein kinase (AMPK) and the complex I of the mitochondrial respiratory chain, whereas the latter effect seems to be more relevant [36,47]. Similar to the in vivo situation, it was shown that metformin directly inhibited androgen production by decreasing CYP17A1 activity [36]. The effect of metformin on androgen production was dose dependent while inhibiting, especially, complex I of the respiratory chain in the mitochondria [36,47]. Interestingly, several studies have examined the electron transport chain function in the brain of children with autism, indicating alterations on different structural and functional levels of the mitochondria. One study examined eight children with autism versus eight healthy controls (4–10 years of age) and reported significantly lower electron transport chain complex activities in the cerebellum, frontal cortex and temporal cortex of the group with autism [48,49]. Another study of temporal lobe brain samples, taken from 20 individuals with autism and 25 controls, found decreased electron transport chain complex I and IV activities and protein content in the group with autism [49,50]. In one study of 15 individuals with autism and 15 controls, the mean activity of the citrate cycle enzyme aconitase was significantly decreased in the cerebellum and temporal lobe in the autism group [49,50,51]. Finally, another study of 14 individuals with autism and 12 controls reported mean reductions in electron transport chain complexes I (31%) and V (36%) activities, as well as in pyruvate dehydrogenase (35%) in the frontal cortex in the autism group, and, in addition, reported a higher mitochondrial DNA (mtDNA) copy number compared to the nuclear DNA in three different mitochondrial genes in the autism group [49,52]. As Metformin directly influences mitochondrial activity, these studies underly the possible usefulness of this pharmaceutical for the treatment of affected subjects with autism. Furthermore, Metformin inhibits oxidative stress [53] and, in addition, it is likely to suggest, that increased oxidative stress upregulates CYP17A1 activity via p38α [9,16,49,54]. Oxidant exposure significantly induced dehydroepiandrosterone production and increased p38α phosphorylation and activation, allowing one to state an association between high androgens and oxidative stress [54]. In detail, p38α inhibition attenuated the H2O2-mediated augmentation of DHEA production with relatively stable 17OHP levels, indicating that activated p38α mediates oxidative-stress-induced 17/20-lyase activation and, in consequence, stimulates androgen synthesis [54]. Interestingly, the effect of oxidative stress on 17alpha-Hydroxylase is far less clear [54], which implies its relevance for autism as 17-alpha Hydroxylase activity was only increased by around 50%, compared to 17/20 Lyase with an increase of 150% in our analyses. Furthermore, these suggestions are in line with a number of studies that indicated the relevance of oxidative stress for autism [49,55,56,57,58,59,60,61,62,63]. Genetic variations in glutathione-related pathways have been observed in affected subjects with autism [58,64,65,66] and have been correlated in some studies with autistic behavior [48,67,68]. Several case–control studies have reported lower concentrations of reduced glutathione (GSH), higher levels of oxidized glutathione (GSSG) and a decrease in the GSH/GSSG redox ratio in autism [56,57,58]. Furthermore, a lower mitochondrial GSH reserve was implied [48,69]. In addition, in some studies, lower GSH levels [70] and markers of increased oxidative stress [71] have been correlated with the severity of autistic traits [48]. Yet, these mentioned studies examined the peripheral markers of oxidative stress, including those found in blood and urine [48]. In addition, a number of studies have reported evidence of oxidative stress in post-mortem brain samples from individuals with ASD compared to controls [48,50,52,72,73,74,75,76,77]. Furthermore, the work by Gevi et al. [33] found that the Tryptophan and Purine metabolism was altered in affected subjects with autism, which has an influence on the mitochondrial activity of Melatonin as Melatonin is suggested to reduce oxidative stress [78]. It was indicated that affected subjects with autism transform tryptophan into xanthurenic acid and quinolinic acid (two catabolites of the kynurenine pathway) at the expense of kynurenic acid and, in particular, of melatonin [33]. Gevi et al., therefore, directly implied a large reduction of melatonin concentrations in affected children with autism and, in consequence, higher levels of oxidative stress [33].

To summarize, understanding the mechanisms regulating 17/20 lyase activity seems essential for the understanding of hyperandrogenic disorders, such as autism, and for the design of selective 17/20 lyase inhibitors, as this step seems mainly affected [16]. A CYP17A1 deficiency can be used as a mirror image concerning the alterations of steroid hormones while enhancing our understanding of the role in affected subjects with autism. To conclude, we suggest that the increased androgens are a result of oxidative stress and mitochondrial dysfunction, as discussed [49]. Pharmaceuticals addressing mitochondria such as Metformin might be a valid therapeutic opportunity for affected subjects with autism [49]. Studies directly elucidating markers of oxidative stress and steroid hormones might give further hints to decipher the enigma of autism.

## Figures and Tables

**Table 1:** Steroid hormone metabolites in gender-segregated comparison of autistic to control children (mean ± SEM); dehydro (DH), tetrahydro (TH), hydroxy (OH).

Urinary Steroid Hormone Metabolites	Boys			Girls		
[μg/mmol creatinine]	Autistic	*p*<	Control	Autistic	*p*<	Control
Androsterone	88.2 ± 10.9	<0.01	49.5 ± 7.1	80.4 ± 21.9	0.06	52.9 ± 11.6
Etiocholanolone	57.7 ± 8.0	<0.01	28.8 ± 4.1	65.0 ± 17.1	0.07	35.8 ± 10.1
DHEA	18.8 ± 8.8	0.11	4.6 ± 1.0	14.5 ± 7.9	0.18	4.7 ± 2.5
Androstendione	5.0 ± 1.2	0.08	2.7 ± 0.4	6.7 ± 2.4	0.06	2.4 ± 0.6
Testosterone	2.8 ± 0.5	<0.01	1.1 ± 0.2	0.8 ± 0.1	0.01	0.6 ± 0.1
TH-11β-DH-Corticosterone	14.5 ± 1.3	<0.01	10.9 ± 1.0	10.7 ± 1.6	0.88	9.5 ± 1.2
TH-Corticosterone	14.6 ± 1.1	<0.01	10.5 ± 0.8	9.7 ± 1.1	0.93	8.8 ± 1.5
5α-TH-Corticosterone	43.7 ± 7.8	<0.01	26.6 ± 2.6	18.9 ± 2.6	0.63	21.3 ± 4.5
Cortisol	11.5 ± 1.6	<0.01	7.2 ± 0.7	6.3 ± 0.7	0.04	4.5 ± 0.6

## Data Availability

Data is available on qualified request to the corresponding author.

## Supplementary Materials

The following supporting information can be downloaded at: https://www.mdpi.com/article/10.3390/life12060867/s1, Table S1: Characteristics of the clinical cohort according to DSM-IV with no significant difference for BMI and age between children with autism and healthy controls.
