# Supplementary material for: How Is CYP17A1 Activity Altered in Autism? A Pilot Study to Identify Potential Pharmacological Targets
Source: Life (Basel). 2022 Jun 10;12(6):867. doi: 10.3390/life12060867 (PMC9225657; doi:10.3390/life12060867)
Supplement: Supplementary file 1 [file life-12-00867-s001.zip › life-1723503-supplementary.pdf]

Supplementary Table S1. Characteristics of the clinical cohort according to DSM-IV with no significant difference for BMI and age between children with autism and healthy controls.

	**n**	**BMI [kg/m²]**	**Age [y]**
		Boys	
Kanner syndrome	21	20.4 ± 1.3	13.6 ± 0.8
Asperger syndrome	20	18.2 ± 0.7	15.3 ± 0.6
Atypical	7	18.1 ± 1.0	13.1 ± 1.3
Total diseased	48	19.1 ± 0.6	14.2 ± 0.5
Healthy	48	18.6 ± 0.3	14.3 ± 0.5
		Girls	
Kanner syndrome	8	16.9 ± 1.0	14.4 ± 1.5
Asperger syndrome	7	18.2 ± 1.2	13.0 ± 1.9
Atypical	1	17.9	15.0
Total diseased	16	17.5 ± 0.7	13.8 ± 1.0
Healthy	16	17.2 ± 0.8	13.2 ± 0.8
